# Supplementary material for: Thermochemical Micro‐Explosion for Prompt Thrombolysis via Proximal Injection of Liquid Alkali Metal
Source: Adv Sci (Weinh). 2026 Jul 3;13(54):e00039. doi: 10.1002/advs.202600039 (PMC13334603; doi:10.1002/advs.202600039)
Supplement: Supplementary file 1 [file ADVS-13-e00039-s008.docx]

**Supplementary Materials**

**Thermochemical Micro-Explosion for Prompt Thrombolysis via Proximal Injection of Liquid Alkali Metal**

**Authors:** Xin Liao^1,5^, Yi Hou^2,5^, Jie Zhang^2^, Bo Wang^3^, Minghui Guo^2^, Hua Qu^4,*^, Wei Rao^2^^,*^, Jing Liu^1,2,*^

**Affiliations:**

1. School of Biomedical Engineering, Tsinghua University, Beijing 100084, China

2. State Key Laboratory of Cryogenic Science and Technology, Technical Institute of Physics and Chemistry, Chinese Academy of Sciences, Beijing 100190, PR China

3. Institute of Materials Research & Center of Double Helix, Shenzhen International Graduate School, Tsinghua University, Shenzhen 518055, China

4. Xiyuan Hospital of China Academy of Chinese Medical Sciences, Beijing 100091, China

5. These authors contributed equally: Xin Liao, Yi Hou

* Corresponding Author. Email: jliu@mail.ipc.ac.cn; weirao@mail.ipc.ac.cn; hua_qu@yeah.net

**Contents**

Supplementary Fig. 1 | High-speed imaging for micro-explosion dynamics. (a) Initial state: water-air interface. (b-h) After adding LAM@oil, LAM reacted with water gradually, accompanied by the generation and continuous rupture of fine and dense microbubbles, and the reaction is completed within 180 s.

Supplementary Fig. 2 | Temperature-time profiles of 20 μL LAM@40/80/100 μL oil in the target reaction zone. A. 20 μL LAM@40 μL oil, B. 20 μL LAM@80 μL oil, C. 20 μL LAM@100 μL oil.

Supplementary Fig. 3 | Temperature-time profiles of 5/10 μL LAM@20/40/80 μL oil in the target reaction zone. A. Temperature-time profiles of 5 μL LAM@20/40/80 μL oil in the target reaction zone, B. Temperature-time profiles of 10 μL LAM@20/40/80 μL oil in the target reaction zone.

Supplementary Fig. 4 | Temperature-time profiles of 5μL LAM in the target reaction zone. (n = 3, independent experiments) A. Temperature-time profiles of 5 μL LAM (Individual A), B. Temperature-time profiles of 5 μL LAM (Individual B), C. Temperature-time profiles of 5 μL LAM (Individual C).

Supplementary Fig. 5 | Thrombolytic efficacy evaluation of 1 μL unencapsulated liquid alkali metal LAM: (A, B) Untreated static thrombus model. (C, D) Thrombus after treatment with 1 μL unencapsulated liquid LAM. (E) Thrombus pre-/post-treatment with 1µL LAM. (F) Residual thrombus weight quantification after treatment.

Supplementary Fig. 6 | Comparative analysis of static thrombi before and after LAM@oil treatment.

Supplementary Fig. 7 | Long-term Durability of LAM@oil Therapeutic Efficacy in SD Rat Carotid Artery In Vivo Thrombolysis Experiment. (a) Laser speckle image of SD rats before modeling, with normal blood flow. (b) Bright-field image of SD rats before modeling, with normal blood flow. (c) Laser speckle image of SD rats after establishing the carotid artery thrombosis model, with blood flow occlusion. (d) Bright-field image of SD rats with the carotid artery thrombosis model, with blood flow occlusion. LAM@oil Treatment Group: (e), (i), (n), (r) Laser speckle images of SD rats before modeling, with normal blood flow. (f), (j), (o), (s) Laser speckle images of SD rats after establishing the carotid artery thrombosis model, with blood flow occlusion. (g), (k), (p), (t) Laser speckle images of SD rats immediately after LAM@oil treatment, with restored blood flow patency. (h), (l), (q), (u) Laser speckle images of SD rats 14 days after LAM@oil treatment, with sustained blood flow patency. All images above were acquired using LSBFMS.

Supplementary Fig. 8 | LSBFMS-monitored blood flow in SD rat carotid arteries: Pre-treatment (a–d): Control (saline), UK group, Dimethylsilicone oil group, LAM@oil group Post-treatment (e–h): Corresponding groups at multiple time points. Scale bar: 10 mm.

Supplementary Fig. 9 | Hematoxylin and eosin (H&E) staining of major organs from rats after in vivo thrombolysis with LAM@oil.

Supplementary Fig. 10 | H&E staining of major organs from non-thrombosis SD rats 14 days post-intravascular injection of LAM@oil.

Supplementary Fig. 11 |H&E analysis of vascular sections at arterial reaction sites from non-thrombosis SD rats 14 days post-injection of LAM@oil.

Supplementary Fig. 12 | Hematoxylin-eosin (H&E) staining images of major organs from carotid artery thrombosis rats receiving LAM@oil thrombolytic therapy (2 days-short-term and 14 days-long-term observations).

Supplementary Fig. 13 | Body weight changes of SD rats in the sham control group, LAM@oil thrombolytic therapy group, and untreated model group.

Supplementary Fig. 14 | Evaluation of LAM@oil for femoral artery thrombolysis in rabbits. (a) Laser speckle image of rabbit femoral artery before modeling, with normal blood flow. (b) Laser speckle image of rabbit femoral artery thrombosis model, with blood flow occlusion. (c) Laser speckle image of rabbit femoral artery after thrombolysis with LAM@oil, with restored blood flow. (d) Bright-field image of rabbit femoral artery before modeling. (e) Bright-field image of rabbit femoral artery thrombosis model. (f) Bright-field image of rabbit femoral artery after thrombolysis with LAM@oil.

Supplementary Fig. 15 | Residual silicon content in blood after LAM@oil treatment.

Supplementary Table 1 | Complete blood count (CBC) of rats at day 0: Control vs. LAM@oil.

Supplementary Table 2 | CBC of rats at day 2: Control vs. LAM@oil.

Supplementary Table 3 | CBC of rats at day 14: Control vs. LAM@oil.

Supplementary Table 4 | Hematological tests of carotid artery thrombosis rats after LAM@oil thrombolytic therapy (short-term and long-term observations).

Supplementary Table 5 | Serum biochemical tests of carotid artery thrombosis rats after LAM@oil thrombolytic therapy (short-term and long-term observations).

Supplementary video 1 | Reaction process between LAM@oil and PBS solution (50× speed).

Supplementary video 2 | Interaction of LAM@oil with static thrombus model (50× speed).

Supplementary video 3 | Thrombolytic progression of LAM@oil within a dynamic thrombotic system composed of 3D vascular constructs.

Supplementary video 4 | Chemical ablation phenomena during LAM@oil -thrombus interaction in an in vitro dynamic thrombolysis model.

Supplementary video 5 | Intra-arterial injection of LAM@oil in SD rat in vivo carotid artery thrombolysis model.

Supplementary video 6 | Therapeutic intervention with LAM@oil in SD rat in vivo carotid artery thrombolysis model.

Supplementary video 7 | Therapeutic intervention with dimethylsilicone oil in SD rat in vivo carotid artery thrombolysis model.

Supplementary video 8 | Therapeutic intervention with Urokinase (UK) in SD rat in vivo carotid artery thrombolysis model.

Supplementary video 9 | High-speed imaging for micro-explosion dynamics-initial state.

Supplementary video 10 | High-speed imaging for micro-explosion dynamics-reaction process.

**Fig. S1.** High-speed imaging for micro-explosion dynamics. (a) Initial state: water-air interface. (b-h) After adding LAM@oil, LAM reacted with water gradually, accompanied by the generation and continuous rupture of fine and dense microbubbles, and the reaction is completed within 180 s.

**Fig. S2.** Temperature-time profiles of 20 μL LAM@40/80/100 μL oil in the target reaction zone. A. 20 μL LAM@40 μL oil, B. 20 μL LAM@80 μL oil, C. 20 μL LAM@100 μL oil.

**Fig. S3.** Temperature-time profiles of 5/10 μL LAM@20/40/80 μL oil in the target reaction zone. A. Temperature-time profiles of 5 μL LAM@20/40/80 μL oil in the target reaction zone, B. Temperature-time profiles of 10 μL LAM@20/40/80 μL oil in the target reaction zone.

**Fig. S4.** Temperature-time profiles of 5 μL LAM in the target reaction zone. (n = 3, independent experiments) A. Temperature-time profiles of 5 μL LAM (Individual A), B. Temperature-time profiles of 5 μL LAM (Individual B), C. Temperature-time profiles of 5 μL LAM (Individual C).

**Fig. S5.** Thrombolytic efficacy evaluation of 1 μL unencapsulated liquid alkali metal LAM: (A, B) Untreated static thrombus model. (C, D) Thrombus after treatment with 1 μL unencapsulated liquid LAM. (E) Thrombus pre-/post-treatment with 1µL LAM. (F) Residual thrombus weight quantification after treatment.

**Fig. S6.** Comparative analysis of static thrombi before and after LAM@oil treatment.

**Fig. S7.** Long-term Durability of LAM@oil Therapeutic Efficacy in SD Rat Carotid Artery In Vivo Thrombolysis Experiment. (a) Laser speckle image of SD rats before modeling, with normal blood flow. (b) Bright-field image of SD rats before modeling, with normal blood flow. (c) Laser speckle image of SD rats after establishing the carotid artery thrombosis model, with blood flow occlusion. (d) Bright-field image of SD rats with the carotid artery thrombosis model, with blood flow occlusion. LAM@oil Treatment Group: (e), (i), (n), (r) Laser speckle images of SD rats before modeling, with normal blood flow. (f), (j), (o), (s) Laser speckle images of SD rats after establishing the carotid artery thrombosis model, with blood flow occlusion. (g), (k), (p), (t) Laser speckle images of SD rats immediately after LAM@oil treatment, with restored blood flow patency. (h), (l), (q), (u) Laser speckle images of SD rats 14 days after LAM@oil treatment, with sustained blood flow patency. All images above were acquired using LSBFMS.

**Fig. S8.** LSBFMS-monitored blood flow in SD rat carotid arteries: Pre-treatment (A-D): Control (saline), UK group, Dimethylsilicone oil group, LAM@oil group Post-treatment (E-H): Corresponding groups at multiple time points. Scale bar: 10 mm.

**Fig. S9.** Hematoxylin and eosin (H&E) staining of major organs from rats after in vivo thrombolysis with LAM@oil.

**Fig. S10**. H&E staining of major organs from non-thrombosis SD rats 14 days post-intravascular injection of LAM@oil.

**Fig. S11.** H&E analysis of vascular sections at arterial reaction sites from non-thrombosis SD rats 14 days post-injection of LAM@oil.

**Fig. S12.** Hematoxylin-eosin (H&E) staining images of heart, liver, spleen, lung and kidney (from left to right) from carotid artery thrombosis rats receiving LAM@oil thrombolytic therapy (2 days for short-term observation and 14 days for long-term observation).

**Fig. S13**. Body weight changes of SD rats in the sham control group, LAM@oil thrombolytic therapy group, and untreated model group.

**Fig. S14.** Evaluation of LAM@oil for femoral artery thrombolysis in rabbits. (a) Laser speckle image of rabbit femoral artery before modeling, with normal blood flow. (b) Laser speckle image of rabbit femoral artery thrombosis model, with blood flow occlusion. (c) Laser speckle image of rabbit femoral artery after thrombolysis with LAM@oil, with restored blood flow. (d) Bright-field image of rabbit femoral artery before modeling. (e) Bright-field image of rabbit femoral artery thrombosis model. (f) Bright-field image of rabbit femoral artery after thrombolysis with LAM@oil.

**Fig. S15.** Residual silicon content in blood after LAM@oil treatment.

**Table S1.** Hematological tests of carotid artery thrombosis rats after LAM@oil thrombolytic therapy (short-term and long-term observations).

**Table S2.** Serum biochemical tests of carotid artery thrombosis rats after LAM@oil thrombolytic therapy (short-term and long-term observations).

**Table S3.** Complete blood count (CBC) of rats at day 0: Control vs. LAM@oil.

**Table S4.** CBC of rats at day 2: Control vs. LAM@oil.

**Table S5.** CBC of rats at day 14: Control vs. LAM@oil.

**Nak reaction mechanism:**

Widespread adoption in academic and industrial contexts: As summarized in the review by Leonchuk et al.^[1]^, this eutectic ratio is the most commonly used composition in NaK-based research and applications. Its popularity stems from its unique combination of favorable properties that other ratios cannot simultaneously achieve, making it the benchmark composition for exploring NaK’s biomedical and material potential.

Superior phase stability at ambient and physiological temperatures: The eutectic composition exhibits exceptional phase stability, maintaining a liquid state at temperatures as low as -12.65 °C (as confirmed by the Na-K phase equilibrium diagram and physical property data in the review)^[2]^. This is far below room temperature (25 °C) and physiological temperature (37 °C), ensuring that the alloy remains fluid during storage, handling, and in vivo application. In contrast, non-eutectic ratios (e.g., Na:K = 50:50 or 80:20) have higher melting points (ranging from 63.5 °C to 97.8 °C for pure K and Na, respectively) and are prone to solidification under ambient conditions, which would compromise injectability and reaction uniformity.

The reactions of pure Na, pure K, and the eutectic NaK alloy with water follow the following equations:

- Pure Na: 2Na+2H_2_O=2NaOH+H_2_↑	(1)
- Pure K: 2K+2H_2_O=2KOH+H_2_↑  (2)
- Eutectic NaK (Na₀.₂₂₂K₀.₇₇₈): 2Na_0.222_K_0.778_+2H_2_O=0.444NaOH+1.556KOH+H_2_↑  (3)

The potential of NaK composition tuning for other biomedical scenarios (beyond thrombosis): for example, a higher Na content (e.g., Na:K = 30:70) with lower peak temperature could be explored for mild thermal ablation of superficial tumors; a marginally higher K content (e.g., Na:K = 20:80) with faster reaction kinetics might be adapted for rapid disintegration of dense pathological deposits in degenerative diseases. These extensions, while beyond the scope of our current thrombolytic focus, demonstrate the broader versatility of NaK-based systems, enabled by the clear understanding of element contributions you emphasized.

**Supplementary Videos:**

**Video S1.** Reaction process between LAM@oil and PBS solution (50× speed).

**Video S2.** Interaction of LAM@oil with static thrombus model (50× speed).

**Video S3.** Thrombolytic progression of LAM@oil within a dynamic thrombotic system composed of 3D vascular constructs.

**Video S4.** Chemical ablation phenomena during LAM@oil -thrombus interaction in an in vitro dynamic thrombolysis model.

**Video S5.** Intra-arterial injection of LAM@oil in SD rat in vivo carotid artery thrombolysis model.

**Video S6.** Therapeutic intervention with LAM@oil in SD rat in vivo carotid artery thrombolysis model.

**Video S7.** Therapeutic intervention with dimethylsilicone oil in SD rat in vivo carotid artery thrombolysis model.

**Video S8.** Therapeutic intervention with Urokinase (UK) in SD rat in vivo carotid artery thrombolysis model.

**Video S9.**  High-Speed Imaging for Micro-Explosion Dynamics-Initial state.

**Video S10.** High-Speed Imaging for Micro-Explosion Dynamics-Reaction process.

**References:**

[1] Leonchuk SS, Falchevskaya AS, Nikolaev V, Vinogradov VV. NaK Alloy: Underrated Liquid Metal. J Mater Chem A. 2022;10:22955–22976.

[2] Foust OJ, ed. Sodium-NaK Engineering Handbook. Gordon and Breach, Science Publishers, Inc.; 1978.
